# Mass Vaccination with a New, Less Expensive Oral Cholera Vaccine Using Public Health Infrastructure in India: The Odisha Model

**DOI:** 10.1371/journal.pntd.0002629

**Published:** 2014-02-06

**Authors:** Shantanu K. Kar, Binod Sah, Bikash Patnaik, Yang Hee Kim, Anna S. Kerketta, Sunheang Shin, Shyam Bandhu Rath, Mohammad Ali, Vittal Mogasale, Hemant K. Khuntia, Anuj Bhattachan, Young Ae You, Mahesh K. Puri, Anna Lena Lopez, Brian Maskery, Gopinath B. Nair, John D. Clemens, Thomas F. Wierzba

**Affiliations:** 1 Regional Medical Research Center, Bhubaneswar, Odisha, India; 2 International Vaccine Institute, Seoul, Republic of Korea; 3 Directorate of Health Services, Odisha, India; 4 University of the Philippines Manila, National Institutes of Health, Manila, Philippines; 5 Translational Health Science and Technology Institute, Department of Biotechnology, New Delhi, India; 6 University of California, Los Angeles School of Public Health, Los Angeles, California, United States of America; Massachusetts General Hospital, United States of America

## Abstract

**Introduction:**

The substantial morbidity and mortality associated with recent cholera outbreaks in Haiti and Zimbabwe, as well as with cholera endemicity in countries throughout Asia and Africa, make a compelling case for supplementary cholera control measures in addition to existing interventions. Clinical trials conducted in Kolkata, India, have led to World Health Organization (WHO)-prequalification of Shanchol, an oral cholera vaccine (OCV) with a demonstrated 65% efficacy at 5 years post-vaccination. However, before this vaccine is widely used in endemic areas or in areas at risk of outbreaks, as recommended by the WHO, policymakers will require empirical evidence on its implementation and delivery costs in public health programs. The objective of the present report is to describe the organization, vaccine coverage, and delivery costs of mass vaccination with a new, less expensive OCV (Shanchol) using existing public health infrastructure in Odisha, India, as a model.

**Methods:**

All healthy, non-pregnant residents aged 1 year and above residing in selected villages of the Satyabadi block (Puri district, Odisha, India) were invited to participate in a mass vaccination campaign using two doses of OCV. Prior to the campaign, a *de jure* census, micro-planning for vaccination and social mobilization activities were implemented. Vaccine coverage for each dose was ascertained as a percentage of the censused population. The direct vaccine delivery costs were estimated by reviewing project expenditure records and by interviewing key personnel.

**Results:**

The mass vaccination was conducted during May and June, 2011, in two phases. In each phase, two vaccine doses were given 14 days apart. Sixty-two vaccination booths, staffed by 395 health workers/volunteers, were established in the community. For the censused population, 31,552 persons (61% of the target population) received the first dose and 23,751 (46%) of these completed their second dose, with a drop-out rate of 25% between the two doses. Higher coverage was observed among females and among 6–17 year-olds. Vaccine cost at market price (about US$1.85/dose) was the costliest item. The vaccine delivery cost was $0.49 per dose or $1.13 per fully vaccinated person.

**Discussion:**

This is the first undertaken project to collect empirical evidence on the use of Shanchol within a mass vaccination campaign using existing public health program resources. Our findings suggest that mass vaccination is feasible but requires detailed micro-planning. The vaccine and delivery cost is affordable for resource poor countries. Given that the vaccine is now WHO pre-qualified, evidence from this study should encourage oral cholera vaccine use in countries where cholera remains a public health problem.

## Author Summary

Cholera – an acute life-threatening diarrheal illness – continues to disrupt public health in resource poor countries. The devastating outbreaks in Haiti and Zimbabwe – to name just two of many occurrences – calls for the use of available oral cholera vaccines as an additional tool in the arsenal of cholera control measures. An oral cholera vaccine (Shanchol) has been licensed in India since 2009; however, there has only been limited use of this vaccine in government public health programs. A vaccination campaign using 2 doses of Shanchol was conducted in Odisha, India, during May and June, 2011, where 31,552 persons (61% of the target population) received the first dose and 23,751 of them completed their second dose. The vaccine delivery cost was $0.49 per dose. Through our findings and experience, we discuss the organization of the cholera vaccination campaign in Odisha, the challenges met for conducting the campaign and the strategies designed to overcome those challenges, and the delivery costs incurred in the use of this vaccine, the first of its kind, in a public health setting. We believe that evidence from this study is of significant interest and use to policymakers from countries where cholera remains a public health problem.

## Introduction

Cholera continues to pose a public health threat in resource- poor countries. Estimates suggest that 1.4 billion people are at risk for cholera, with 2.8 million cases and 91,000 deaths occurring annually in cholera-endemic countries worldwide [1]. The devastating and prolonged outbreaks of cholera in Haiti (with 682,475 cases and 8,328 deaths as of October 9, 2013), and in Zimbabwe (with >98,000 cases and 4,000 deaths as of July 2009) [2], [3] demand the use of cholera vaccine as an additional tool in the arsenal of cholera control measures. Given the potential of cholera outbreaks to disrupt health systems, the World Health Organization (WHO) recommends that available oral cholera vaccines (OCVs) be used in conjunction with other preventive and control strategies in areas where the disease is endemic and in areas at risk for outbreaks [3].

Until recently, Dukoral - a monovalent, whole cell killed vaccine with recombinant B sub unit cholera toxin (WC/rBS) had been the only WHO-prequalified OCV available for use. However, due to its relatively high cost (about US$ 5.3/dose for public sector), the use of Dukoral has been primarily limited to travelers from developed and higher income countries. A bivalent, killed, whole-cell OCV that was reformulated by the International Vaccine Institute (IVI) was licensed in India in 2009 based on results from a phase III trial in Kolkata, India. This vaccine, called Shanchol, is safe and confers 65% protective efficacy after 5 years post-vaccination, as measured by the reduction in the number of culture-confirmed cholera cases [4]. Shortly after its licensure, recommendations were made at a national-level policymakers' meeting in Delhi, India [5] to conduct pilot vaccine introductions in endemic areas such as one in Orissa (now Odisha) in India, distributing the two-dose OCV by utilizing the existing public health infrastructure. It is worth to mention that about 514 million people are at risk for cholera, with 834,000 cases and 25,000 deaths occurring annually in India [1].

Odisha, which is adjacent to the Bay of Bengal, is one of the most natural disaster-prone states in India [6]. It is severely affected by seasonal floods and droughts, creating conditions that facilitate the spread of cholera. Almost every year, from May to November, coastal areas in Odisha experience cyclones and floods. During this time, outbreaks of diarrheal illness often due to cholera occur [7]–[10]. In a ten year review of reported and published cholera cases in India, Odisha had the highest number of affected individuals in cholera outbreaks and had reported cholera in seven out of ten years [11]. A three-year diarrheal disease surveillance study (2004–2006) conducted by the Regional Medical Research Center (RMRC), in Bhubaneswar, Odisha, which involved taking stool specimens or rectal swabs from admitted cases on a weekly basis at three hospitals, found that, among a total of 1,551 stool and rectal collected swab samples, up to 17.3% tested positive for cholera [12]. A large outbreak in tribal districts (Koraput, Kalahandi, and Rayagada) of Odisha between August and September, 2007, was caused by a new hybrid strain which is believed to cause more severe disease [8].

While Shanchol is licensed in India, there has only been limited use of the vaccine and no documentation on how the vaccine would be deployed using government public health resources. The primary objective of this study was to evaluate the feasibility, acceptability and costs of using a less expensive oral cholera vaccine delivered through the government's public health infrastructure. We describe the organization of the vaccination campaign in Odisha, the challenges met for conducting the campaign, and the strategies designed to overcome those challenges. We also present vaccine coverage by age groups and sex, and the delivery costs incurred in the use of this vaccine, the first of its kind, in a public health setting.

## Methods

### Ethics statement

The study protocol was approved by all of the following: the Health and Family Welfare Department, Government of Odisha; the Human Ethical Committee of the Regional Medical Research Center (RMRC) in Bhubaneswar, Odisha; the Health Ministry Screening Committee, Government of India; and the Institutional Review Board of the International Vaccine Institute in Seoul, Korea. This study was registered as number NCT01365442 with clinicaltrials.gov. Informed consent was obtained both at the community level through meetings with community leaders, and at the individual level through verbal informed consent just before vaccination.

### Study site, population and the public health system

The state of Odisha in India, with a population of about 37 million, is composed of 30 districts where each district is divided into 3–26 blocks [13]. In the public health system, blocks are further sub-divided into sub-centers (the lowest public health unit) of various sizes. Each sub-center is supported by a midwife nurse. In the villages within each sub-center, public health activities are also supported by volunteers called ASHA (Accredited Social Health Activist) and AWW (Anganwadi workers).

**Figure 1:**
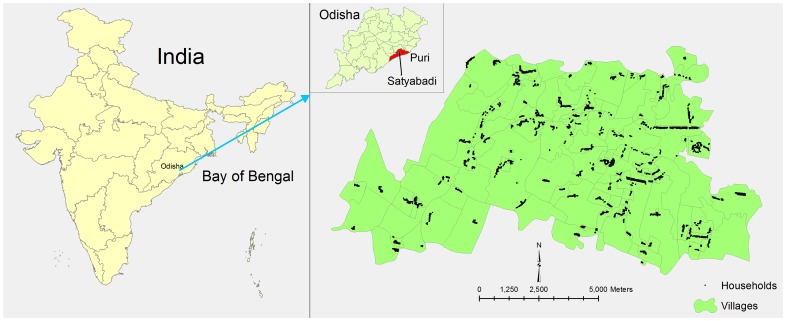
Study area in Satyabadi block, Odisha, India.

The Directorate of Health Services (DHS), in consultation with RMRC, suggested conducting a mass vaccination in Satyabadi block of Puri District because DHS data from 2005 to 2007 suggested that this block had the highest number of severe diarrhea cases, presumably due to cholera. Diarrhea cases increase during the monsoon season (usually July to September) every year in the study area. We therefore decided to complete the vaccination before the start of rainy season. Of the 19 sub-centers in Satyabadi block, 10 sub-centers with 145 villages and hamlets encompassing approximately 50,000 people were targeted for vaccination (Figure 1). Four supervisors, thirteen midwives, forty-nine ASHA and sixty-seven AWW (133 health providers in total) implement immunization activities – both regular vaccination and campaigns -in the catchment area of the selected ten sub-centers.

### Census

A baseline census to determine the target population was carried out from February 9 to April 2, 2011. Trained project staff made house-to-house visits to collect demographic (e.g., age, sex) and social (e.g., marital status, educational level) information of all members in each household. In addition, data on primary occupation, access to water, sanitation, and hygiene practices for each household was also collected. A unique number was assigned to each household and all of its members. From the census database, a household identification (ID) card was generated containing information on the total number of household members, name, age, sex and marital status of each member in that particular household. Laminated ID cards were then distributed to each household by the community health volunteers (ASHA and AWW). The household members were requested to bring the ID cards at the time of vaccination.

### Vaccine and cold chain

Shanchol is a modified bivalent killed whole cell-based oral cholera vaccine given in two doses at least 14 days apart [4]. The antigens are provided in a 1.5 ml liquid formulation in a 2.5 ml glass vial. The vaccine was presented in single-dose vials contained in small cardboard boxes. During vaccination, a cardboard box was opened and the removed vial was shaken before its liquid contents were directly poured into the vaccinee's mouth. This was at times followed by a drink of clean water; no buffer was required.

### Micro-planning

A detailed micro-plan was developed in consultation with health volunteer and community leaders to identify the location of each vaccination booth. Each booth was selected after ensuring that no villager traveled more than 10 to 15 minute by foot to reach a booth. The number of vaccination days, staffing of immunization booths (their working hours and supervision structure), and transport of vaccines and ice-packs from the central storage facility to each booth, were assessed during a series of meetings with public health officials at the state, district, block, and sub-center levels.

We assessed the vaccine storage capacity and ice-pack production/storage facilities at the state, district, and block levels to identify any potential cold-chain gaps during a vaccination campaign. This assessment was done by meeting with public health officials and by making site visits before the campaign. Based on the number of booths and cold chain boxes/vaccine carriers required at each booth, we calculated the projected number of ice-pack required for each day during each phase of the campaign.

We also conducted various community mobilization activities to raise awareness about the importance of the campaign and to encourage participation. Before the campaign, meetings were organized to inform community leaders and health care providers at the state, district and block levels about cholera, the oral cholera vaccine profile and the upcoming mass vaccination activities in the area. Prior to and throughout the campaign, information was disseminated within the study area using local newspapers, posters, leaflets, banners and mobile announcements (‘miking’). In addition, a door-to-door outreach campaign was also carried out by the local health volunteers.

### Mass vaccination

All healthy, non-pregnant (as ascertained by verbal screening) residents from the study area aged 1 year and above were invited to participate in the mass vaccination. A vaccination registry (vaccination record book) with pre-printed information for each participant from the baseline census database was used to record dosing status. Considering the public health implications, individuals who wished to receive the vaccine but lived outside the study area were also given an opportunity to participate in the vaccination campaign. A separate vaccination registry was maintained to record vaccination data for persons who could not be found in the vaccination record book, either because they were from outside the study area, or because they had not been accounted for during the baseline census survey. A vaccination card (different from the household ID card described earlier) was issued to each participant, whether they were from the study area or not, at the time of administration of the first dose. Each participant was requested to bring his/her vaccination card at the time of second dose administration. Similarly, persons who took a first dose in the second round, were asked to present to any of the two public health facilities in the area after 14 days to receive a second dose on two fixed dates.

### Vaccine coverage

The information from the vaccination record book was doubly entered into a password-protected computerized database developed using Microsoft Visual FoxPro 7.0. Vaccine coverage for each dose was ascertained as a percentage of the eligible censused population (one year and older). Drop-out rates in the second dose were calculated based on 1^st^ dose of vaccination. Vaccine coverage was also stratified by age groups (children: 1–5, older children: 6–17, adults: 18–60, and older adults: 61+ years) and sex (male, female). Vaccine wastage rate was estimated by comparing the delivered number of doses with the vaccine coverage for the censused population.

### Delivery costs

The input cost items required for the vaccination campaign, along with respective quantities, were identified and listed onsite by a health economist; cost items related to research were excluded. Subsequently, financial receipts and records maintained at the field office were matched against the listed input items to estimate unit costs. In the case of items for which expenditure invoices were not available, the costs were collected by interviewing management and finance staff involved in the mass vaccination campaign. At the end, to confirm that all the expenses were included, financial costs collected at the field were cross-verified with the itemized expenditure reports submitted by RMRC to IVI.

The primary cost items included special activities conducted for the mass vaccination such as: vaccine price, freight and shipment, storage and transport, cold-chain maintenance and logistic support, sensitization meetings and various social mobilization activities, training of staff, incentives and travel support for vaccinators, supervisors and cold chain handlers, surveillance activities for the management of adverse events following immunization and vaccine procurement. For the cost estimation, although the vaccine was obtained at a subsidized price of US $1 per dose for this study, we have used US $1.85 per dose, which is the current market price of Shanchol for public health programs in less-developed countries. Only costs of vaccine delivered to target and non-target population and wasted vials were taken into account. Excluded cost items were: staff time spent on program planning, costs of vaccine storage equipment and utilities, and costs of unused vaccines. Similarly, rental costs for training rooms and vaccination booths were excluded because the campaign employed the existing government infrastructure. Cost of waste management was excluded as it was absorbed within existing government waste management system. Costs were presented based on the mean exchange rate between US dollars and Indian National Rupees (1 USD = 46.7 INR) and on data from the International Monetary Fund [14] as of 2011.

## Results

### Study population

A total of 51,865 persons residing in 9,166 households in the study area were enumerated during the baseline census survey (Table 1). After excluding children below one year of age, 51,488 persons were defined as the targeted population for the mass vaccination. The population was predominantly Hindus (98%) with a density of 508 inhabitants per square kilometer (km^2^) and dispersed on small plots of land, approximately 30 km away from the sea (Indian Ocean). The majority of adults (80%) and nearly half of all children (46%) used open field defecation. Two-thirds of the populations were dependent on community tap/hand pump for drinking water. It was observed that bathing and washing clothes/utensils usually took place in ponds distributed around the community (data not collected).

**Table 1:** Individual and household characteristics of the study population in Satyabadi block in Odisha, India.

Details	Number	Percent
Individual characteristics:-		
Total population	51,865	100.0
Education level:		
Illiterate	9,227	17.8
Literate but without formal education	7,725	14.9
Primary school	15,419	29.7
Secondary school	11,201	21.6
Other (high school, graduate, etc.)	8,293	16.0
Household characteristics:-		
Total households	9,166	100.0
Religion of household head:		
Hindu	8,972	97.8
Muslim	194	2.2
Major occupation of the household head:		
Farmer	4,044	44.1
Daily wage laborer	1,849	20.2
Traders/selling goods	622	6.8
Retired	599	6.5
Unemployed	616	6.7
Other (fisherman, service worker etc.)	1,436	15.7
Type of toilet for adults (13 years and above):		
Latrine with cement	1,563	17.0
Latrine without cement	254	2.8
Open field	7,334	80.0
Other	15	0.2
Type of toilet for children (up to 12 years):		
Latrine with cement	669	7.2
Latrine without cement	105	1.2
Open field	4,195	45.8
Other/(No children up to 12 years)	4,197	45.8
Main source of drinking water:		
Own tap/well/hand pump	2,000	21.8
Community tap/well/hand pump	6,745	73.6
Pond water/Other	421	4.6
Is water generally boiled before drinking?		
Always	253	2.8
Sometimes	2,140	23.4
Never	6,765	73.8
Do not know	8	0.1
After defecation, hand wash with:		
Water only	953	10.4
Water and soil/ashes	6,418	70.0
Water and soap	1,795	19.6

### Vaccine and cold chain

A total of 77,000 doses of the vaccine (assuming 80% coverage with first dose, 15% drop out and 5% wastage) were transported from Hyderabad, the capital city of the Indian state of Andhra Pradesh, to Bhubaneswar, the capital city of the Indian state of Odisha. Transport required cold boxes by special delivery van to maintain the cold-chain. Ten single-dose glass vials of Shanchol, each contained within a small cardboard box, were packaged in an outer carton; 54 of these cartons were placed in a thermochol box (dimensions: 0.46 m, 0.38 m, 0.29 m). A seven-cubic meter space was required to store 77,000 vials. Since there were only 4 small refrigerators available (each with a volume of 0.09 cubic meter) at the Primary Healthcare Center (PHC) serving the catchment population, vaccines were stored in a ‘walk-in cooler’ at the State Drug Management Unit at Bhubaneswar. The walk-in cooler temperature was monitored and maintained between +2° to +8°C. The routine Expanded Program on Immunization (EPI) cold boxes were used for transportation of vaccines to the study area on a daily basis during the campaign. Similarly, since ice-pack production and storage facilities were very limited at the PHC level (48 ice-packs per day), we used the ‘walk-in freezer’ facility at the state level to meet the ice-packs requirement for the campaign. At the time of delivery, about 50 vials, each with a small outer cardboard box, could be placed in one routine EPI vaccine carrier. To accommodate more vials in the vaccine carrier, we removed the outer cardboard box while still in the cold room so that about 90 vials could be kept in 1 vaccine carrier. Approximately 450 and 700 ice-packs were required daily for the first and second phases, respectively.

### Micro-planning and mass vaccination

Two phases of the vaccination campaign, each with two dosing rounds (3 vaccination days in each round), were conducted (Table 2) from May 5 to June 4, 2011. Of the 62 vaccination booths that were established in the community, 59 were located in schools and 3 were established in local clubs. We conducted the campaign in 2 phases to overcome the deficit in number of staff, cold boxes and ice-packs that needed to be at each of the vaccination booths. For example, for all 62 booths to operate in a single phase, with at least 5 workers at each booth, 310 workers would have been needed. Each booth was led by a midwife and supported by 5–6 community health workers/volunteers. A total of 260 health workers (midwives and volunteers) were provided with a one-day training session on vaccination. Training was held five times between April 29 and May 3, 2011.

**Table 2:** Micro-planning for the cholera vaccination campaign in Satyabadi block in Odisha, India.

Details	First phase	Second phase
Vaccination days of 1^st^ round	May 5–7, 2011	May 12–14, 2011
Vaccination days of 2^nd^ round	May 26–28, 2011	June 2–4, 2011
Vaccination days for people receiving first dose in 2^nd^ round	June 11 and 18, 2011	June 18 and 25, 2011
No. of Catchment population	∼20,000	∼30,000
No. of sub-center	4	6
No. of booth	23	39
No. of booth-member^1^	7	6
No. of first level supervisor	8	12
No. of second level supervisor	5	5
No. of mobile mini center^2^	8	12
No. of cold boxes (10–20 liters)	12	16
No. vaccine carriers	50	90
No. of ice-packs/day on vaccination days	450	700
Other logistics	Registration log book for in-census and not-in-census, vaccination cards, paper, pen, forceps, waste boxes etc	

1 midwives and volunteers at each booth.

2 To replenish vaccines and ice-packs at each booth.

Each team performed the following activities on vaccination days: screening for eligibility, obtaining verbal consent from each participant, administering vaccine, filling tally sheets and vaccination registration books, monitoring for immediate adverse events for up to 30 minutes, issuing vaccination cards, collecting remaining vaccine vials and wastes (aluminum and rubber lids, used vaccine vials) at the end of each session, and bringing waste back to the designated health facility. Used vaccine vials were destroyed by incineration while other wastes were buried at the PHC; unused vials were donated to the DHS.

Each booth was open daily from 7.00 am to 5.00 pm for three consecutive days in each round. Eight vehicles in the first phase and twelve vehicles in the second phase were used to transport staff, cold boxes with vaccines and ice-packs, and other supplies. Each vehicle was manned by one supervisor. These mobile vans (mini-centers) were also used to replenish vaccines and ice-packs during the campaign.

### Vaccine coverage

A total of 31,552 eligible censused persons (61% of the target population) received the first dose of vaccine and 23,751 (46%) of these completed their second dose, accounting for a 25% drop-out between the two doses. In addition, 4,446 persons who were either not captured during census or were from outside the study area, received the first dose and 2,170 of these completed the second dose. Thus, 55,303 doses of vaccine were delivered to the eligible censused population and 12% of vaccine (6,616 doses) was given to people outside the censused population. An additional 6% of vaccine (3,312 doses) was wasted. The main reasons for wastage were: broken vials, empty vials, spillage, or persons failing to swallow.

Vaccine coverage, stratified by age groups and sex, is shown in Table 3. The highest coverage rate was achieved for 6- to 17-year-olds, while adults below 60 years of age had the lowest coverage. Males had lower coverage for both first and second dose. The lowest drop-out rates were observed among females (23%) and among the 6- to 17- year-olds (21%).

**Table 3:** Vaccine coverage* by age** groups and sex in Satyabadi block, Odisha, India.

	Target population	At least one dose recipients No. (%)	Two dose recipients No. (%)
**Age groups (years)**			
1–5	3,807	2,698 (71)	2,116 (56)
Male	1,937	1,371 (71)	1,068 (55)
Female	1,870	1,327 (71)	1,048 (56)
6–17	11,361	8,817 (78)	6,975 (61)
Male	5,767	4,359 (76)	3,425 (59)
Female	5,594	4,458 (80)	3,550 (63)
18–60	31,171	17,167 (55)	12,467 (40)
Male	15,435	7,475 (48)	5,044 (33)
Female	15,736	9,692 (62)	7,423 (47)
61+	5,149	2,870 (56)	2,193 (43)
Male	2,610	1,512 (58)	1,169 (45)
Female	2,539	1,358 (53)	1,024 (40)
**Sex**			
Male	25,749	14,717 (57)	10,706 (42)
Female	25,739	16,835 (65)	13,045 (51)
**Total**	**51,488**	**31,552 (61)**	**23,751 (46)**

* Coverage is defined as number of people who received vaccine dose(s)/Target population ×100.

** Age at the start of vaccination (May 5, 2011).

### Delivery costs

The total cost of the vaccination program was US$ 149,574 or US$ 2.7 per dose delivered to the target population (Table 4). Vaccine cost at market price (US$1.85) was the largest cost item. Omitting vaccine shipment (from Hyderabad to Bhubaneswar) cost (US$0.04, not shown), the vaccine delivery cost was US $0.49 per dose, or US $1.13 per fully vaccinated person. Vaccines provided to persons outside of the census population were considered a public health good, and the delivery cost per dose was reduced to US $0.44, or US $1.04 per fully vaccinated person while accounting for them.

**Table 4:** Public sector costs (in 2011 prices) of cholera vaccination campaign in Satyabadi block in Odisha, India.

Cost item	Total costs (US$^1^)	Cost/dose (US$)	Total costs (%)
Social mobilization	5,603	0.10	3.75
Vaccine^2^	122,629	2.22	81.99
Vaccine storage and transport	2,081	0.04	1.39
Vaccine administration	15,022	0.27	10.04
AEFI^3^ management	4,237	0.08	2.83
**Total**	**149,574**	**2.71**	**100**

1 *US$ = United States Dollar.*

2 *Vaccine cost includes vaccine wastage and vaccine delivered to non-target population.*

3 *AEFI = Adverse Events Following Immunization.*

## Discussion

This was the first project to be undertaken to collect empirical evidence for mass vaccination campaign using the new, less expensive oral cholera vaccine (Shanchol) in a government-run, public health program. This was also the first opportunity to conduct a mass vaccination campaign using Odisha's public health system, which is already capable of supporting mass vaccination campaigns against other diseases, like polio. However, unlike the usual polio campaigns, in which the target population is roughly 13% (0- to 5-year-olds), the catchment population for this cholera campaign was almost the entire community. In addition, in terms of outreach comparisons, community residents were well aware of the polio vaccine and their health providers/volunteers were generally trained on its delivery, as polio campaigns are conducted regularly. In contrast, our cholera campaign had to raise awareness of a new vaccine. Our campaign also differed from polio campaigns in terms of the number of booths: whereas only 25 vaccination booths are generally established in our catchment area for polio campaigns, we established 62 booths to cover a wider population for the cholera vaccine. This had implications for additional requirements of human resources and of vaccine carriers/ice-packs.

In terms of cold-chain infrastructure comparisons, the polio vaccine volume is small (2 drops or 0.1 mL) in multi-dose vials compared to the 1.5 mL single-dose vials used for Shanchol; therefore, the space required for cholera vaccine storage and transportation is greater. Further, conducting a mass vaccination campaign for an entire community poses considerable challenges for the public health infrastructure, particularly human resources and cold chain capacity at the peripheral health facilities. For example, there were only 129 midwives and volunteers in the catchment area, while we needed a total of 161 booth members for the first phase and 234 for the second phase of the campaign. Fortunately, since we conducted the mass vaccination in a catchment area of half of a primary health care (PHC) facility, additional human resources could be mobilized from the other half of the same PHC and from nearby PHCs. Typically, for other mass vaccination campaigns, like polio campaigns, two to three persons at each vaccination booth would accomplish the entire set of activities in Odisha, India, and in most of other developing country settings. However, this project required additional manpower to obtain verbal informed consent, perform registration, and issue vaccination cards, all activities which are not typically done during vaccination campaigns conducted in a public health setting. This requirement for additional manpower also increased the delivery costs, a phenomenon which was observed in an earlier study [15].

The limited capacity to produce and store ice-packs at the peripheral health care facilities was overcome by the availability of cold chain facilities located at the central level, which were within a two hours' drive from the study area. Further, receiving the vaccines in two lots also made it easier to store them. During administration, one has to remove the aluminum lid first, shake the vial well, and then remove the rubber lid before pouring the vaccine into the participant's mouth. Simpler packaging of the vial, in plastic tubes, for example, and production of multi-dose vials without outer cardboard box would greatly facilitate its storage and delivery and should be considered for future production and use of this vaccine.

According to the vaccine package insert, the temperature must be maintained between +2° to +8°C; thus, storage of vaccines and their distribution with cold chain maintenance posed substantial challenges under hot and humid weather conditions, with temperatures reaching up to 42°C on vaccination days. Since this is an inactivated vaccine, recommendations from the manufacturer or from the regulatory authority on waiver of strict cold chain requirements (e.g., during vaccine delivery at a minimum) would greatly facilitate its use in future campaigns. A similar recommendation for the use of killed whole cell/recombinant B-subunit (WC/rBS) cholera vaccine, Dukoral, was made based on vaccine delivery experiences in Indonesia in 2005 [16]. The recent approval of a meningitis vaccine (MenAfrivac) to be stored and transported without ice-packs/refrigeration for four days could also facilitate similar recommendation for Shanchol in the future [17].

Additional vaccine carriers and larger-sized cold boxes (10 to 20 liters in capacity) were mobilized from nearby PHCs and central level institutions. The use of a phased approach during the implementation of the campaign greatly helped to overcome the challenges due to human resources and cold chain infrastructure and should be considered in future large-scale vaccination campaigns. The absence of buffer preparation and co-administration with this new vaccine also minimized associated logistic challenges that were observed in previous studies with WC/rBS [15], [16], [18].

Our findings suggest that mass vaccination using two doses of OCV, where almost the entire community is targeted, is doable but requires detailed micro-planning, additional human resources, modifications in cold-chain capacity and modifications in the number and the location of vaccination booths. In our case, we showed that it was feasible to install vaccine booths within an average distance of 267–283 meters from the households. According to our booth location plan, 1,087 meters was the maximum possible distance a person had to walk to reach a booth. This micro planning was made in an effort to maximize coverage.

Nonetheless, the level of vaccine coverage achieved during this campaign (46–61%) was lower compared to the coverage observed (59–83%) during previous studies with other OCVs [16], [18], [19]. The lower vaccine coverage reported here may be due to several reasons. Due to ongoing routine weekly public health activities (e.g., immunization days, nutrition days etc.) conducted by the PHC, we could not implement the campaign beyond three days for each round. In comparison, vaccination campaigns with other OCVs were usually held for about10 days [16], [18], [19]. In addition, we speculate that a low turn-out in the hot and humid weather (with temperatures reaching up to 42°C) was a factor associated with the relatively lower coverage in this study. Further, since adults (18–60 years) were observed to have the least coverage, innovative strategies (e.g., operating vaccination booths until late in the evening or early in the morning) to catch this group should be considered for future campaigns. This was noted as well for the previous studies concerning the use of WC/rBS [15]. At the end of each vaccination day, unused vials with cold chain and wastages from vaccination needed to brought back to the PHC; therefore, we had to stop vaccination by 5 pm to allow for unused vial collection and transportation. Invariably, there were people still queuing for vaccination at the end of the day, but booths needed to be closed. They were requested to visit again the following day.

We also observed that some participants did not like the ‘taste and smell’ of this vaccine, which they described as ‘fishy’ or ‘rotten egg’ in nature. Vaccination days were from Thursdays to Saturdays in each round of each phase, and since Thursdays are ‘complete vegetarian days’ for Hindus in the study area, the vaccine's taste and smell could potentially influence lower coverage in this particular community. However, further studies are needed to substantiate or refute this taste-influence hypothesis in this community. In addition, in spite of raising cholera vaccine awareness during community outreach programs, the perception that “vaccines are only for children” remained prevalent in the communities (data not collected); this could explain, at least in part, the lower coverage observed among adults compared to children. Since the oral cholera vaccine was being introduced for the first time, it was regarded as a ‘new’ vaccine to both the providers and the community residents and this perception could have also influenced the lower coverage. The relatively high drop-out rates could have been due to adverse weather conditions and unpleasant taste/smell of vaccine, which merit further investigation. We also observed that people who did not take any dose of vaccine tended to be older, were male and belonged to high socio-economic status of the community (data not shown).

The public sector vaccine delivery cost of $0.49 per dose excluding vaccine freight and shipment was similar to the vaccine delivery cost estimate for a campaign-based delivery from the “WHO comprehensive Multi-Year Plans Guidelines for EPI Vaccines in 2006” [19] and is within the range of estimates from previous studies using other OCV [15], [18], [20]. The delivery cost was higher than the $0.09 and $0.23 per dose estimates reported from campaigns in Vietnam in 1998 [20] and in a refugee camp in Uganda in 1997 [18]. When the delivery costs are readjusted to 2011 price based on country inflation consumer prices [21], the delivery cost in Odisha is still higher than that in Vietnam ($0.23) but lower than that in Uganda ($0.56). Further, the delivery cost estimate was lower than the $0.94 estimated in 2003/2004 ($1.97 in 2011 prices) per dose in Beira, Mozambique, where the very high vaccine transportation cost was a factor [15].

A cholera vaccination economic model using incidence estimates for high-risk populations in India from a recent global burden study [1] estimated a cost effectiveness ratio of $785 per DALY averted for programs targeted to ages 1 year and above in South East Asia Region, where cholera vaccine coverage is assumed at 80% and 50% of measles vaccine coverage for populations 1–14 and 15+ years, respectively [22]. Applying these estimates, cholera vaccination would be considered “very cost effective” based on WHO criteria [23]. Without cholera incidence data, it is not possible to estimate the Odisha-specific cost-effectiveness of vaccination. However, we observed that the vaccine coverage for two-dose recipients was 62% in 1–14 years and 41% in 15+ years which are, respectively, more than 80% and 50% of measles vaccine coverage (76%) in Odisha in 2010–2011 [24].

Since we conducted the vaccination campaign in a cholera-endemic setting, we believe that our methods and our findings provide a model that may be extrapolated to other endemic settings in India – a country which accounts for an estimated 30% of global cholera burden - and beyond. Given that the vaccine is now WHO pre-qualified, evidence from this study is of significant interest and use to policymakers from countries where cholera remains a major public health problem. The vaccine could be a viable, affordable, and effective tool in public health programs to control cholera in these countries.
